# Classification of Genicular Artery Anatomic Variants Using Intraoperative Cone-Beam Computed Tomography

**DOI:** 10.1007/s00270-023-03411-3

**Published:** 2023-03-22

**Authors:** Tyler E. Callese, Lucas Cusumano, Karen D. Redwood, Scott Genshaft, Adam Plotnik, Jessica Stewart, Siddharth A. Padia

**Affiliations:** 1 Division of Interventional Radiology, University of California, Los Angeles, Los Angeles, USA; 2 University of California, Los Angeles, Los Angeles, USA; 3 Department of Radiology, David Geffen School of Medicine at UCLA, 757 Westwood Plaza, Los Angeles, CA 90095 USA

**Keywords:** Osteoarthritis, Embolization, Arterial interventions, Genicular artery embolization

## Abstract

**Purpose:**

Genicular artery embolization (GAE) is a new treatment option for symptomatic knee osteoarthritis. Genicular arterial anatomy is complex with limited published reports. This study describes the genicular artery anatomy utilizing intraprocedural cone-beam computed tomography (CBCT) during GAE.

**Materials and Methods:**

This retrospective single-center study was approved by the institutional review board. All patients who underwent GAE between May 2018 and April 2022 were reviewed. Patients with a technically adequate CBCT were included in the analysis. CBCTs were analyzed to determine the presence, course, and branching patterns of the genicular arteries.

**Results:**

A total of 222 patients underwent GAE and 205 (92%) were included for analysis. The descending genicular artery was present in 197 (96%) CBCTs with two branches in 152 (77%). The superior medial genicular artery (SMGA) was present in 186 (91%), superior lateral genicular artery (SLGA) in 203 (99%), inferior medial genicular artery (IMGA) in 195 (95%), inferior lateral genicular artery (ILGA) in 196 (95%), and median genicular artery (MGA) in 200 (97%). Four unique branching patterns were identified: common origin of SLGA and MGA (115, 56%), unique origins (45, 22%), trifurcation of SLGA, SMGA, and MGA (32, 15.5%), and common origin of SMGA and MGA (12, 6%). The recurrent ascending tibial was identified in 156 (76%) CBCTs and superior patellar artery in 175 (85%) CBCTs.

**Conclusion:**

Genicular artery anatomy is complex with numerous common variants. CBCT is a powerful adjunct in GAE to rapidly identify target vessels for embolization and potentially decrease the risk of nontarget embolization.

## Introduction

Genicular artery embolization (GAE) is a rapidly emerging treatment option for symptomatic mild-to-moderate knee osteoarthritis (OA) with promising early trials [1–5]. Approximately 45% of patients will develop symptomatic OA in their lifetime [6]. Chronic chondral inflammation in OA stimulates neoangiogenesis and sensory nerve growth contributing to knee pain. GAE selectively decreases areas of hypervascularity, decreasing pain [1, 3]. GAE is expected to become increasingly common as patients’ active lifespans increase and they wish to defer knee arthroplasty [7].

GAE is a technically challenging procedure due to the highly variable arterial supply to the knee. Given that genicular artery intervention has been uncommon in the past, there is a paucity of published reports describing genicular artery anatomy. Many of the published reports are on a small number of patients, or on cadaveric specimens [8–10]. The purpose of this study was to describe genicular artery anatomy utilizing intraoperative cone-beam computed tomography (CBCT) during GAE for knee pain secondary to OA.

## Materials and Methods

### Patients

This single-center retrospective observational study was approved by the institutional review board and informed consent was waived. All patients who underwent genicular artery embolization for symptomatic knee osteoarthritis between May 2018 and April 2022 were reviewed.

Procedures were performed at a single academic tertiary care center in the ambulatory setting. Inclusion criteria for analysis included patients with a technically adequate intraoperative CBCT. Exclusion criteria in this analysis included patients without CBCT or without a technically adequate CBCT. Reasons for technical inadequacy included incorrect bolus timing, motion, or artifact.

### Technique

GAE was performed by one of three board certified interventional radiologists following previously described technique [5].

CBCT are routinely performed in all GAE cases per institutional protocol [5]. CBCT were performed through an ipsilateral CFA sheath or contralateral catheter using a power injector containing a mixture of 2:1 Omnipaque (GE healthcare, Chicago, Illinois) 350 mg Iodine/mL to saline. Injection rate was 3 mL/sec for 20 secs (total of 60 cc contrast delivered) utilizing a 10 s delay and 10 s CBCT acquisition. Multiplanar reformats and rotational 3D reconstructions were performed on in-line workstation and exported to PACS. Imaging was done using a Philips Allura Suite (Best, Netherlands).

Following CBCT, areas of hypervascularity identified on DSA and CBCT and corresponding radiopaque pain indicators were selectively embolized following previously described technique [5].

#### Genicular Artery Anatomy

**Fig. 1:**
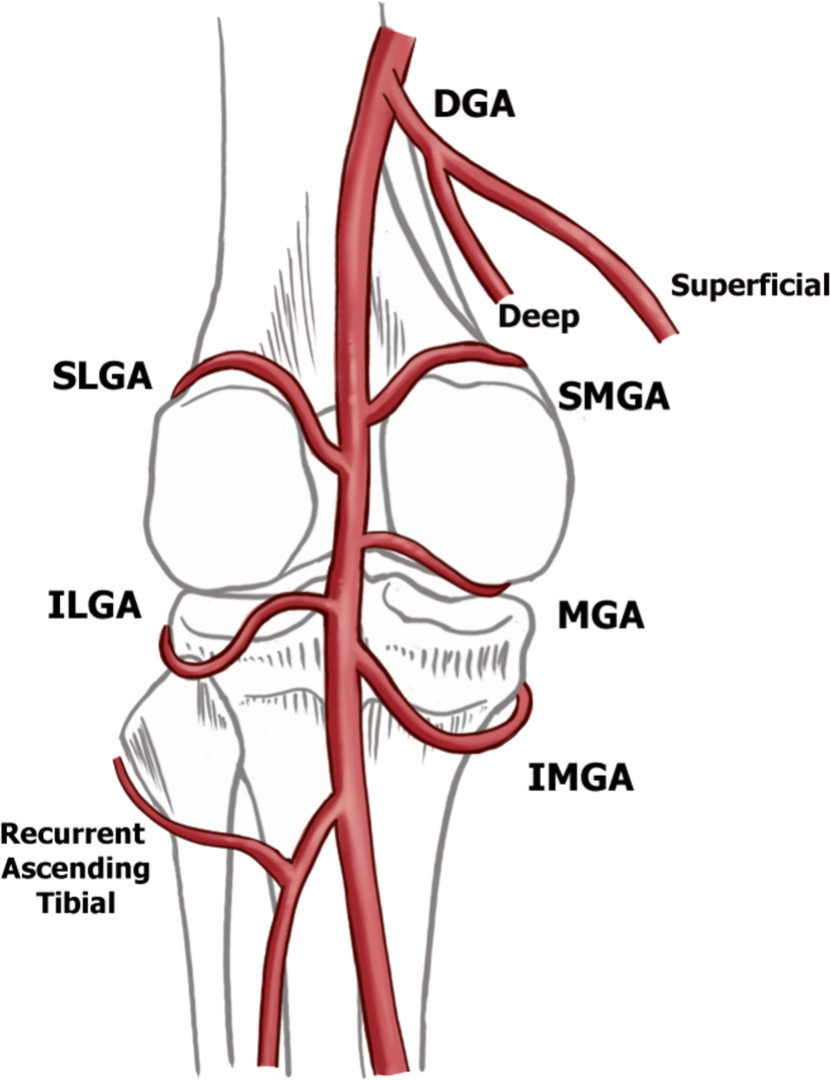
Schematic of the genicular arteries

There are eight arteries supplying the knee joint implicated in GAE (Fig. 1): descending genicular artery (DGA), superior lateral (SLGA), superior medial (SMGA), median (MGA), inferior medial (IMGA), inferior lateral (ILGA), superior patellar (SPA), and recurrent anterior tibial arteries (RATA).

**Fig. 2:**
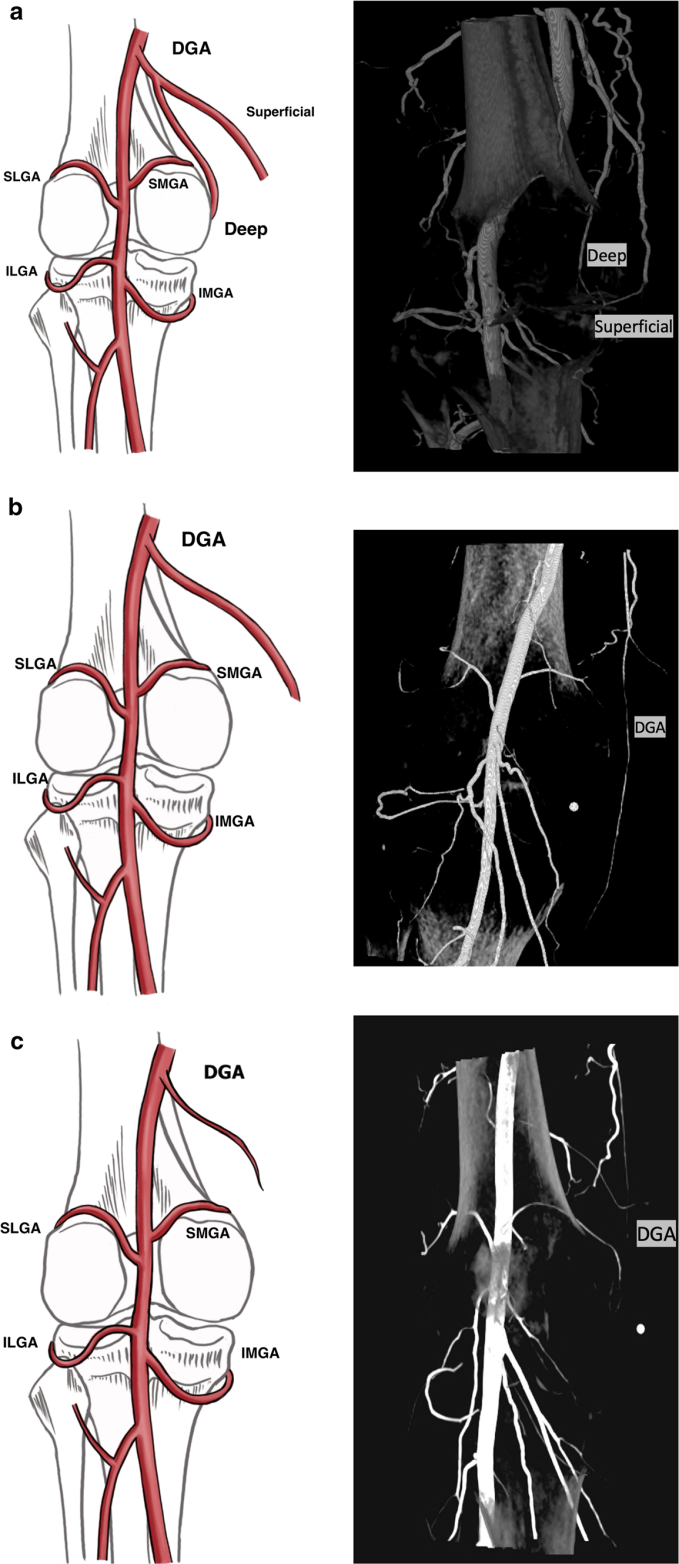
Descending genicular artery branching variants with intraoperative contrast-enhanced cone-beam computed tomography correlate. Four branching patterns were identified for the descending genicular artery (DGA), including **a** two branches (77%), **b** no branching (17%), **c** diminutive (5%), and absent (1%)

The superior aspect of the knee joint is supplied by the DGA, arising off the femoral artery, which commonly branches into deep (supplying the joint) and superficial (myocutaneous) branches (Fig. 2). The SPA has a variable origin from the DGA or directly from the femoral artery and takes a characteristic serpiginous course in the anterior knee to the superior pole of the patella and is implicated in patellofemoral compartment OA synovial changes [11]. The superior lateral and medial compartments are supplied by the SLGA and SMGA, respectively. A small caliber MGA supplies the cruciate ligaments and synovium and is often below the resolution of the conventional CT. It is important to preserve during GAE to prevent nontarget embolization of the cruciate ligaments. The inferior knee is supplied by the IMGA and ILGA and RATA (Fig. 1).

#### Analysis

CBCTs were independently reviewed by two interventional radiologists. Genicular artery anatomy was evaluated on each CBCT for the presence, origin, course, and branching anatomy of the DGA, SPA, SLGA, SMGA, MGA, ILGA, IMGA, and RATA. Descriptive statistics were used to compare genicular artery anatomy across the patient cohort.

## Results

A total of 222 patients underwent GAE for symptomatic knee osteoarthritis, 205 (92%) met inclusion criteria and were included in analysis. Seventeen patients were not included due to the absence or technical inadequacy of CBCT. Descriptive patient characteristics are provided in Table 1.

**Table 1:** Baseline characteristics

Total patients (*n*)	222
Age (years, mean, range)	70.0 (44–95)
Weight (kg, mean, range)	81.9 (44–164)
Access site (mean)	74% ipsilateral
Treated knee	55% right
Arteries embolized (mean, st dev)	1.8 ± 0.79

### Descending Genicular Artery

Four branching patterns were identified for the DGA including: two branches (77%), single artery without branching (17%), diminutive (5%), and absent (1%) (Fig. 2). The two branch DGA variant describes a superficial and a deep branch. The superficial branch supplies soft tissues and muscle in the superomedial knee. The deep branch perfuses the medial knee compartment. A single DGA without branching describes a vessel arising off the superficial femoral artery coursing medially and supplying the soft tissues of the superomedial knee. Diminutive DGA describes a small caliber vessel.

### Superior Genicular Arteries

**Fig. 3:**
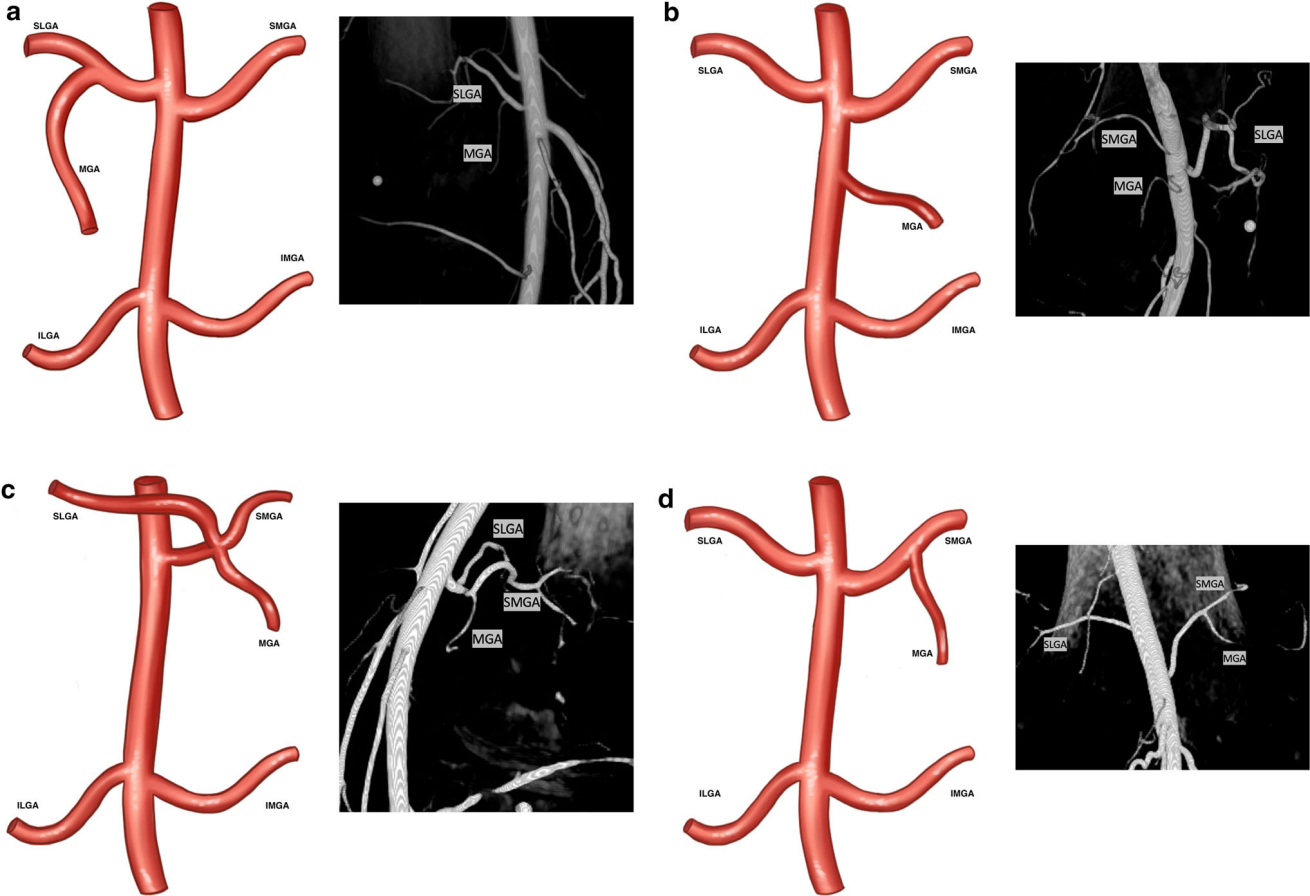
The four types of genicular artery branching with intraoperative contrast-enhance cone-beam computed tomography correlate. Type 1 (**a**, 56.1%)) describes a common trunk of the MGA and SLGA. Type 2 (**b**, 21.9%) describes independent origins of all branches off the popliteal artery. Type 3 (**c**, 15.6%) describes a common trunk of the MGA, SLGA and SMGA. Type 4 (**d**, 5.9%) describes a common trunk of the MGA and SMGA

Four branching patterns were identified in the superior genicular arteries (Fig. 3). Type 1 (Fig. 3a), a common trunk of the MGA and SLGA, was the most common variant seen 56.1% of cases (Table 2). Type 2 (Fig. 3b) includes independent origins of the SLGA, SMGA, and MGA off the popliteal artery and was seen in 21.9% of cases (Table 2). Type 3 describes a common trunk of the MGA, SLGA and SMGA and was seen in 15.6% of cases (Table 2), Type 4 describes a common trunk of the MGA and SMGA (Fig. 3d) and was present in 5.9% of cases (Table 2). Only one case was unclassified (0.5%).

**Table 2:** Comparison of current study to previous reports

Branching pattern of genicular arteries	Current study (*n *= 205 intraoperative CBCT)	Sigary et al. (*n* = 204 cadaver limbs)	O’Grady et al. (*n* = 20 cadaver limbs)
Common trunk: SLGA + MGA	115 (56.1%)	31 (!5%)	4 (20%)
Independent origins	45 (21.9%)	57 (28%)	9 (45%)
Trifurcation: SLGA + MGA + SMGA	32 (15.6%)	20 (10%)	1 (5%)
Common trunk: (SMGA + MGA)	12 (5.9%)	45 (22%)	5 (25%)
Other	1 (0.5%)	31 (15%)	1 (5%)

*CBCT* cone-beam computed tomography, *SLGA* superior lateral genicular artery, *MGA* median genicular artery, *SMGA* superior medial genicular artery

### Inferior Genicular Arteries

Two branching patterns were identified in the inferior genicular arteries: independent origins of the IMGA and ILGA (99.5%) genicular arteries and common trunk of the IMGA and ILGA (0.5%).

### Superior Patellar Artery

**Fig. 4:**
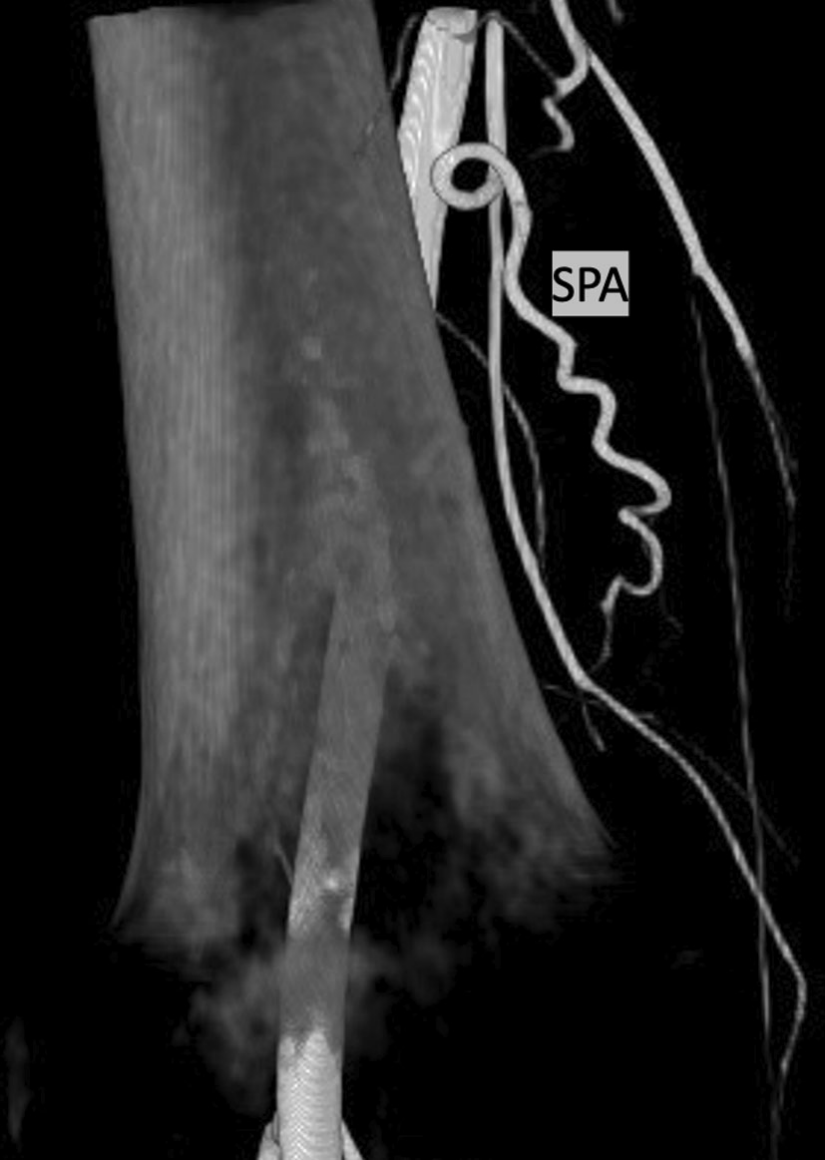
Intraoperative contrast-enhanced cone-beam computed tomography of the superior patellar artery

The SPA (Fig. 4) was identified in 175 patients (85%) and perfused the joint space in 175 (100%) of cases where it was identified. The origin of the artery was beyond the field of view in 84 (48%) of CBCTs.

### Recurrent Anterior Tibial Artery

**Fig. 5:**
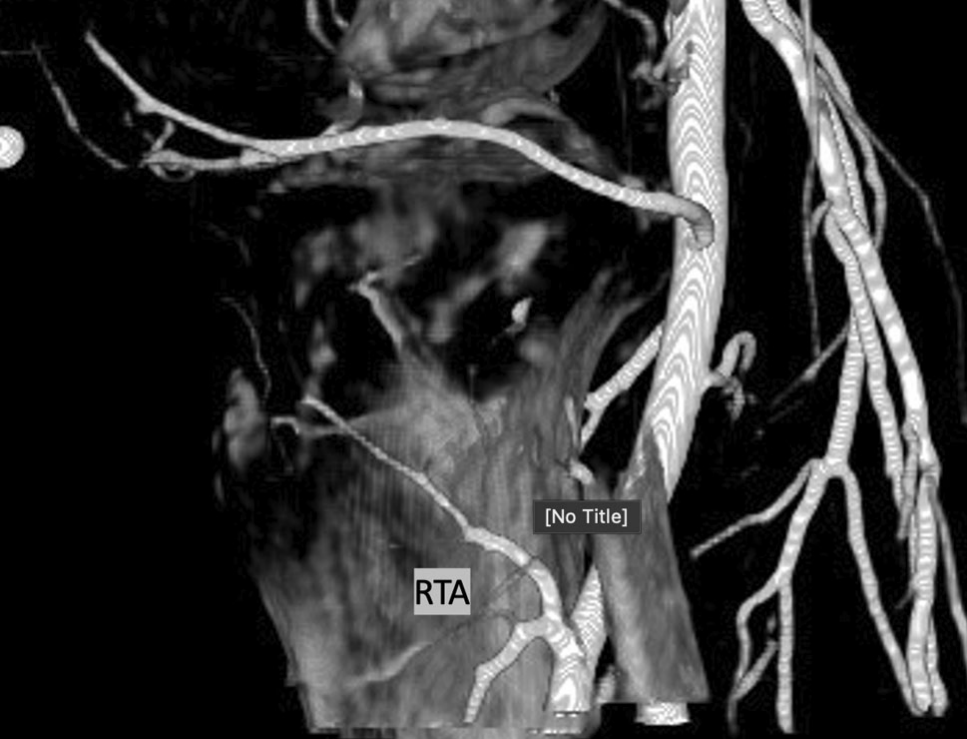
Intraoperative contrast-enhanced cone-beam computed tomography of the recurrent tibial artery

The RATA (Fig. 5) was identified in 156 patients (76%) and perfused the joint space in 121 cases (78%) (Fig. 2). The origin was beyond the field of view in 67 (43%) CBCTs.

## Discussion

There is a need for treatment options for patients with moderate-to-severe knee osteoarthritis in whom deferring or delaying total knee arthroplasty is preferred. Genicular artery embolization (GAE) is an emerging treatment option with promising results from recent trials [1–5]. Genicular arterial anatomy is complex and variable and there is a paucity of published reports describing anatomic variants. Thorough understanding of the anatomy is essential to maximize clinical response and minimize risk of nontarget embolization. The current anatomic study describes genicular arterial anatomy utilizing intraoperative contrast-enhanced cone-beam computed tomography (CBCT).

Intraoperative CBCT is an essential adjunct to conventional angiography and CT angiography. It allows for quick identification of all relevant arteries supplying the affect knee compartment, decreases risk of nontarget embolization, and is higher resolution, uses less contrast, and is more convenient for patients (does not require separate outpatient CTA visit) compared to conventional CT angiography. Compared to standard DSA, laminar flow dynamics are not as much of a concern in identifying arterial anatomy in CBCT, since it involves a prolonged 10 s acquisition during continuous contrast injection.

There are a limited number of published reports describing genicular artery anatomy. Sighary et al. [10] performed genicular artery dissection on 212 cadaver lower limbs. They identified three types of DGA branching and six types of genicular artery branching (4% were not classified) and did not report superior patellar or recurrent anterior tibial arteries. O’Grady et al. [9] performed genicular artery dissection on 20 cadaver lower limbs. They identified five types of genicular artery branching and did not report DGA or recurrent anterior tibial arteries. Comparison of the current study with previous reports is summarized in Table 2.

Cadaveric studies are an important contribution to the growing GAE literature; however, these findings do not necessarily translate to clinical and angiographic findings. Compared to DSA, CBCT is higher resolution and makes small caliber vessels and anastomoses readily identifiable. A previous report on genicular artery angiographic anatomy did not identify the MGA or its common branching variants [8]. This may be technique related or due to it being below the resolution of DSA. Additionally, this highlights one of the limits of single-plane projections in DSA vs. multiplanar CBCT. Similar to advancements in technique for prostate artery embolization, CBCT is essential in GAE to delineate complex arterial anatomy, readily identify target vessels, and mitigate risk of nontarget embolization [12].

In this study, four genicular artery branching variants were identified: Type 1: Common trunk of SLGA and MGA, Type 2: Independent origins, Type 3: Common trunk of the SLGA, MGA, and SMGA, and Type 4: Common trunk of the MGA and SMGA. It is not entirely clear why there is a difference in variant incidence.

## Conclusion

Genicular artery anatomy is complex with several common variants. The present study of genicular arterial anatomy describes the common anatomic arterial variants supplying the knee. CBCT is a powerful adjunct in GAE to rapidly identify target vessels for embolization and potentially decrease the risk of nontarget embolization.
